# Mechanisms involved in an increment of multimodal excitability of medullary and upper cervical dorsal horn neurons following cutaneous capsaicin treatment

**DOI:** 10.1186/1744-8069-4-59

**Published:** 2008-11-19

**Authors:** Kuniya Honda, Junichi Kitagawa, Barry J Sessle, Masahiro Kondo, Yoshiyuki Tsuboi, Yoshiyuki Yonehara, Koichi Iwata

**Affiliations:** 1 Department of Oral and Maxillofacial Surgery, Nihon University School of Dentistry, 1-8-13 Kandasurugadai, Chiyoda-ku Tokyo, 101-8310, Japan; 2 Department of Physiology, Nihon University School of Dentistry, 1-8-13 Kandasurugadai, Chiyoda-ku Tokyo, 101-8310, Japan; 3 Department of Oral Physiology, Faculty of Dentistry, University of Toronto, 124 Edward Street, Toronto, Ontario M5G 1G6, Canada; 4 Division of Functional Morphology, Dental Research Center, Nihon University School of Dentistry, Tokyo 101-8310, Japan; 5 Division of Applied System Neuroscience Advanced Medical Research Center, Nihon University Graduate School of Medical Science, 30-1 Ohyaguchi-Kamimachi Itabashi, Tokyo 173-8610, Japan

## Abstract

**Background:**

In order to evaluate mechanisms that may underlie the sensitization of trigeminal spinal subnucleus caudalis (Vc; the medullary dorsal horn) and upper cervical spinal cord (C1-C2) nociceptive neurons to heat, cold and mechanical stimuli following topical capsaicin treatment of the facial skin, nocifensive behaviors as well as phosphorylation of extracellular regulated-kinase (pERK) in Vc and C1-C2 neurons were studied in rats.

**Results:**

Compared to vehicle application, capsaicin application to the lateral facial skin produced 1 hour later a flare in the skin, and also induced significantly greater nocifensive behaviors to heat, cold or mechanical stimulus of the lateral facial skin. The intrathecal (i.t.) injection of the MEK inhibitor PD98059 markedly attenuated the nocifensive behaviors to these stimuli in capsaicin-treated rats. Moreover, the number of pERK-like immunoreactive (pERK-LI) cells in Vc and C1-C2 was significantly larger following the heat, cold and mechanical stimuli in capsaicin-treated rats compared with vehicle-treated rats. The number of pERK-LI cells gradually increased following progressive increases in the heat or mechanical stimulus intensity and following progressive decrease in the cold stimulus. The ERK phosphorylation in Vc and C1-C2 neurons was strongly inhibited after subcutaneous injection of the capsaicin antagonist capsazepine in capsaicin-treated rats.

**Conclusion:**

The present findings revealed that capsaicin treatment of the lateral facial skin causes an enhancement of ERK phosphorylation in Vc and C1-C2 neurons as well as induces nocifensive behavior to heat, cold and mechanical simulation of the capsaicin-treated skin. The findings suggest that TRPV1 receptor mechanisms in rat facial skin influence nociceptive responses to noxious cutaneous thermal and mechanical stimuli by inducing neuroplastic changes in Vc and C1-C2 neurons that involve in the MAP kinase cascade.

## Background

Thermal allodynia or hyperalgesia to heat, cold or mechanical stimuli can be produced by peripheral inflammation or peripheral nerve injury [1,2]. Capsaicin is an inflammatory irritant and a specific excitant of C- and small-diameter Aδ-fibers innervating peripheral tissues [3-5]. It is well known that capsaicin binds to the transient receptor potential (TRP) vanilloid 1 (TRPV1) channel and induces cation influx in peripheral nerve fiber terminals [6-8]. These receptors are also activated by heat stimulation of peripheral tissues. Strong heat stimulus opens the TRP channels and the cation influx occurs in the nerve fiber terminals, resulting in the generation of action potentials. Both C- and small-diameter Aδ-fiber terminals can be sensitized after capsaicin application to peripheral tissues and their response threshold to heat decreases; conformational changes in the TRPV channel protein are thought to be involved in the sensitization of these channels [6-11]. It is well known that thermal and mechanical hyperalgesia or allodynia are induced in capsaicin-treated skin following sensitization of the C- and small-diameter Aδ-fiber terminals [3-5,12,13]. The capsaicin administration frequently causes the formation of a flare in capsaicin-treated regions, suggesting that C- or Aδ-fibers are activated and that the axon reflex is produced by capsaicin, resulting in plasma extravasation and subsequently flare formation and thermal allodynia in the capsaicin-treated skin [14-16].

Topical application of capsaicin to the facial skin also causes flare formation in the skin and increases heat sensitivity in the capsaicin-treated skin [17]. A high population of trigeminal ganglion (TG) neurons expresses TRP and families of TRPV1 and TRPA1 channel proteins, and some of them also express TRPM8 channel protein [18]. The population difference of each TRP channel in TG neurons is thought to affect the functional differences in processing of heat, cold and mechanical noxious sensory information in the orofacial region. These findings raise the possibility that thermal- and mechano-receptors can become hypersensitive to thermal and mechanical stimuli after capsaicin treatment. However, the mechanisms underlying the sensitization of cold-, heat- and mechano-receptors after capsaicin treatment is not fully understood.

Recent intracellular neuronal recording studies have shown that some nociceptive neurons in the spinal dorsal horn (DH) respond to noxious heat, cold and mechanical stimuli and have specific morphological features; most of these neurons are located in the superficial laminae of the spinal DH [19]. The orofacial inflammation or nerve injury causes a strong activation of trigeminal ganglion neurons such as an increase in the background activity and evoked responses to mechanical or thermal stimulus [20,21]. The barrage of action potentials from the trigeminal nerve fibers is conveyed to trigeminal spinal subnucleus caudalis (Vc; the medullary dorsal horn) and C1-C2 neurons resulting in the significant increase in their excitability. It has also been reported that Vc nociceptive neurons responding to heat, cold and mechanical stimuli are frequently encountered in temporomandibular joint-inflamed rats [22]. These multimodal nociceptive neurons in the DH or Vc are thought to be involved in sensory abnormalities to heat, cold and mechanical nociception following inflammation or peripheral nerve injury [19,22]. It is very important to clarify mechanisms underlying sensory abnormalities following peripheral and central sensitization in order to develop new approaches to treat patients with allodynia or hyperalgesia after inflammation or nerve injury.

The extracellular signal-regulated kinase (ERK) is known as one of the mitogen-activated protein kinases (MAPKs) [23,24]. The ERK in dorsal root ganglion, spinal dorsal horn and Vc neurons is phosphorylated within 10 min following peripheral noxious stimuli [25-28]. The number of phosphorylated ERK (pERK)-immunoreactive cells increased in the DRG and spinal DH as there was an increase in the noxious stimulus intensity [25-29]. Recently, it has been reported that the ERK is phosphorylated in many neurons in the Vc and C1-C2 within 5 min following noxious stimuli [30]. These findings suggest that the activation of neurons following noxious stimuli of the trigeminal structures is reflected by the phosphorylation of ERK in Vc and C1-C2 neurons. These results also indicate that the ERK phosphorylation is a good indicator of nociceptive neurons activated by peripheral noxious stimuli in the trigeminal system.

Therefore, we decided to evaluate mechanisms that may underlie the sensitization of Vc and C1-C2 nociceptive neurons to heat, cold and mechanical stimuli following topical capsaicin treatment of the rat's facial skin by assessing, nocifensive behaviors as well as pERK in Vc and C1-C2 neurons.

## Results

### Flare formation

**Figure 1:**
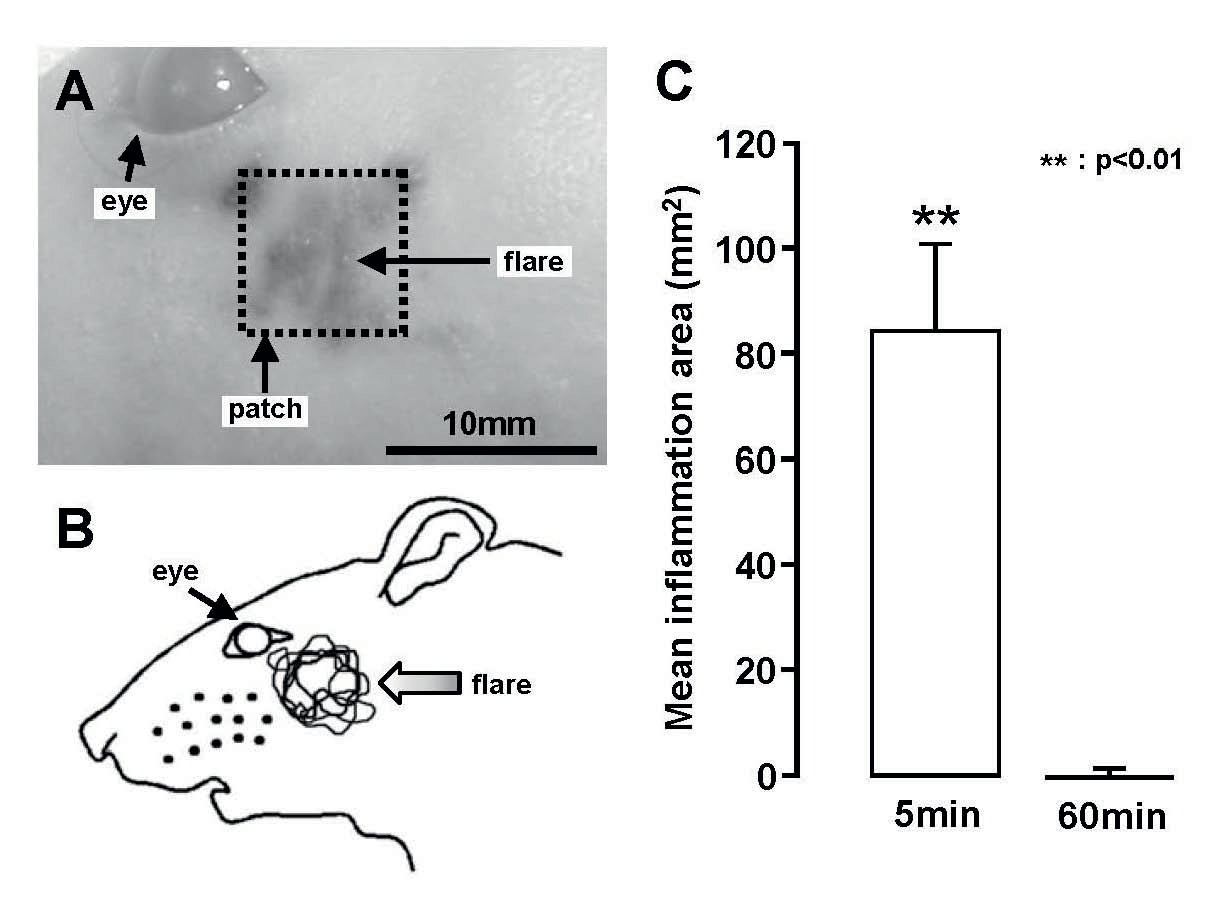
**Flare formation in the facial skin following capsaicin application to the lateral facial skin**. A: Photograph of the lateral facial skin. The flare was observed within the dotted square in the lateral facial skin. B: Outlines of each flare in the lateral facial skin following capsaicin treatment. C: Mean area of the flare in the lateral facial skin at 5 min and 60 min after the removal of 1-hour capsaicin patch. **: p < 0.01.

One hour after placing the capsaicin patch on the lateral facial skin, formation of a flare was apparent as an area of blue staining on the lateral facial skin (Fig. 1A and 1B). However, no flare formation was induced in the face after vehicle patch placement (data not shown). The area of the flare was significantly larger at 5 min after removal of the 1-hour capsaicin patch compared to that at 60 min after the removal of the 1-hour capsaicin patch at which time no flare was apparent (Fig. 1C).

### Nocifensive behaviors

**Figure 2:**
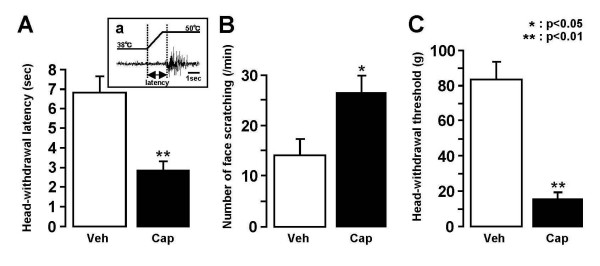
**Nocifensive behaviors to heat, cold or mechanical stimulus of the lateral facial skin**. A: Mean head-withdrawal latency to heating of the skin, Aa: EMG activity recorded from the splenius capitis muscle during heating of the skin, B: Mean number of face-scratching episodes following topical administration of acetone to the skin, C: Mean head-withdrawal threshold to mechanical stimulus of the skin. *: p < 0.05, **: p < 0.01. Veh: vehicle for capsaicin, Cap: capsaicin.

The head-withdrawal latency to heat stimuli or mechanical and the face-scratching frequency after topical application of acetone to the lateral face were significantly affected by capsaicin treatment. The mean head-withdrawal latency to heat stimuli of the face in capsaicin-treated rats was significantly shorter than that in vehicle-treated rats (capsaicin: 2.9 ± 0.4 s, n = 5; vehicle: 6.8 ± 0.8 s, n = 5, p < 0.01) (Fig. 2A). Similarly, the number of face-scratching episodes was significantly larger in capsaicin-treated rats compared to that in vehicle-treated rats (capsaicin: 26.8 ± 3.4/min, n = 5; vehicle: 14.2 ± 3.4/min, n = 5, p < 0.05), as illustrated in Fig. 2B. Mean head-withdrawal threshold to mechanical stimuli of the face was significantly shorter in capsaicin-treated rats than that in vehicle-treated rats (capsaicin: 16.0 ± 4.1 g, n = 5; vehicle: 84.0 ± 9.8 g, n = 5, p < 0.01), as illustrated in Fig. 2C.

### Effect of i.t. administration of PD98059 on nocifensive behavior

**Figure 3:**
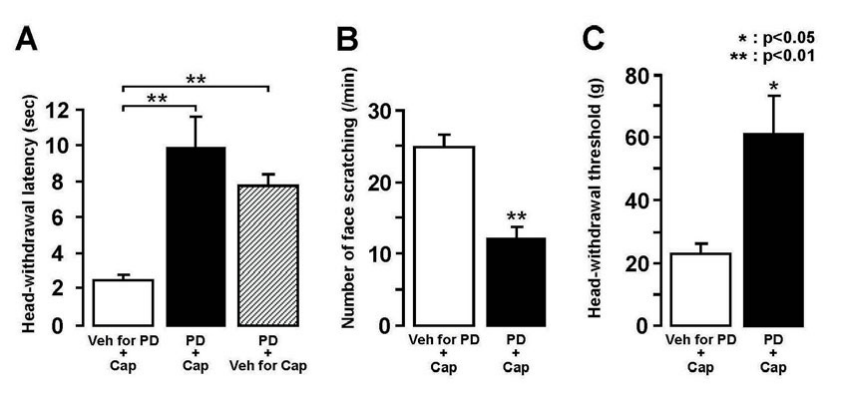
**Effect of PD98059 i.t. injection on the heat, cold and mechanical stimuli-induced nocifensive behavior in capsaicin-treated rats**.A: Mean head-withdrawal latency to heat stimulus of the skin. B: Mean number of face-scratching episodes following acetone application on the skin. C: mean head-withdrawal threshold to mechanical stimulus of the skin. *: p<0.05, **: p<0.01. PD: PD98059, Veh for PD: vehicle for PD98059, Cap: capsaicin, Veh for Cap: vehicle for capsaicin.

The effect of i.t. administration of the MEK inhibitor PD98059 was tested on the heat, cold or mechanical-induced nocifensive behaviors. One week after continuous i.t. infusion of PD98059 or vehicle for PD98059, heat-, cold- or mechanical-induced nocifensive behavior was studied in the capsaicin-treated rats(Fig. 3). We could not observe any spontaneous behavioral changes during i.t. PD98059 injection in the capsaicin-untreated rats. The head-withdrawal latency to heat stimuli of the face after capsaicin treatment in rats with PD98059 i.t. injection was significantly longer compared to that with vehicle i.t. injection (Fig. 3A). The number of face-scratching episodes induced by acetone administration in capsaicin-treated rats with PD98059 i.t. injection was significantly smaller compared to that in vehicle injected rats (Fig. 3B). Furthermore, the head-withdrawal mechanical threshold after capsaicin treatment in rats with PD98059 i.t. injection was significantly higher compared to vehicle-injected rats (Fig. 3C).

### pERK-LI cells in Vc and C1-C2

**Figure 4:**
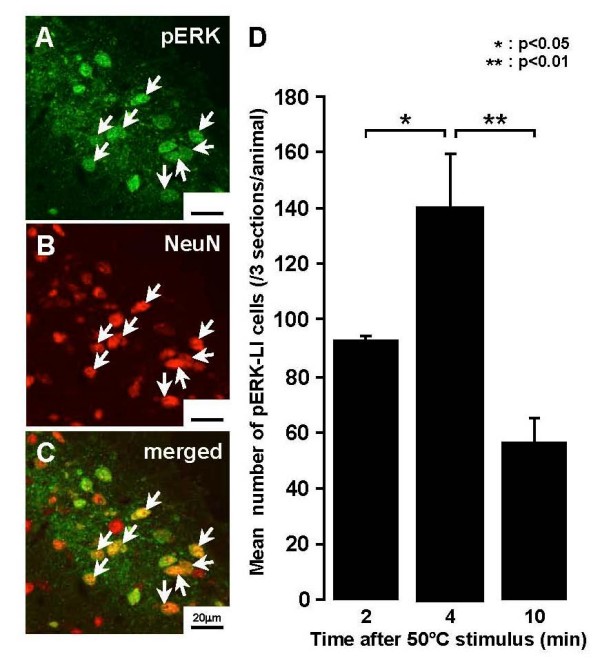
**Photomicrographs of pERK-LI cells and the time course in the number of pERK-LI cells in Vc after heating of the facial skin**. A: pERK-LI cells (green), B: NeuN positive cells (red), C: pERK-LI and NeuN positive cells (yellow); arrows indicate pERK-LI cells and NeuN positive cells in A-C. D: Time course in the number of pERK-LI cells after heat stimulation of the skin in capsaicin-treated rats. *: p < 0.05, **: p < 0.01.

**Figure 5:**
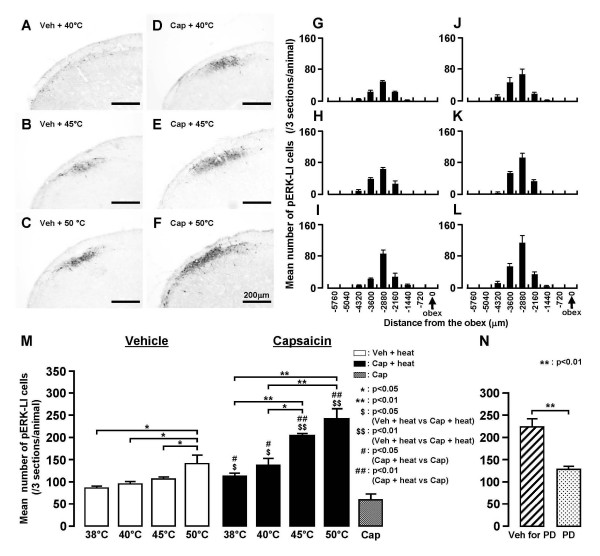
**pERK-LI cells in Vc after graded heat stimulus of the lateral facial skin in rats with capsaicin or vehicle application to the skin**. A-F: Photomicrographs of Vc following 40, 45 and 50°C stimuli of the face in vehicle- (A-C) or capsaicin-treated rats (D-F). G-L: Rostro-caudal distribution of pERK-LI cells in Vc and C1-C2 following graded heat stimulus of the skin in vehicle- (G-I) and capsaicin-treated rats (J-L). M: Mean number of pERK-LI cells in Vc and C1-C2 following graded heat stimulation of the skin in vehicle- (open columns) and capsaicin-treated (solid columns) rats. N: Mean number of pERK-LI cells in Vc and C1-C2 after heating (50°C) of the skin in rats receiving MEK inhibitor PD98059 or vehicle i.t. injection. *: p < 0.05, **: p < 0.01, $: p < 0.05 (Veh + heat vs. Cap + heat), $$: p < 0.01 (Veh + heat vs. Cap + heat), #: p < 0.05 (Cap + heat vs. Cap), ##: p < 0.01 (Cap + heat vs. Cap). Veh: vehicle for capsaicin, Cap: capsaicin, PD: PD98059, Veh for PD: vehicle for PD98059.

**Figure 6:**
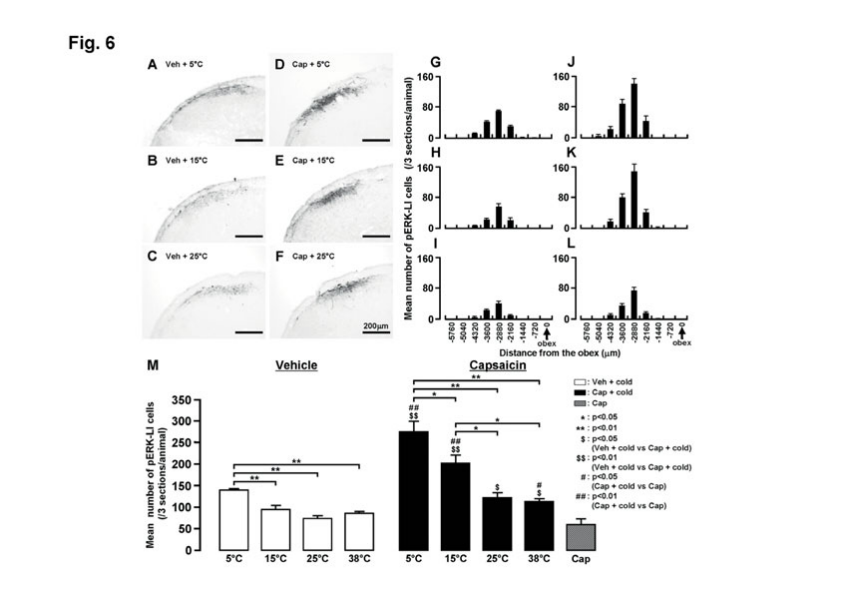
**pERK-LI cells in Vc after graded cold stimulus of the lateral facial skin in rats with capsaicin or vehicle application to the skin**. A-F: Photomicrographs of Vc following 5, 15 and 25°C stimuli of the skin in vehicle- (A-C) or capsaicin-treated (D-F) rats. G-L: Rostro-caudal distribution of pERK-LI cells in Vc and C1-C2 following graded cold stimulus of the skin in vehicle- (G-I) and capsaicin-treated rats (J-L). M: Mean number of pERK-LI cells in Vc and C1-C2 following graded cold stimulus of the skin in vehicle- (open columns) and capsaicin-treated (solid columns) rats. *: p<0.05, **: p<0.01, $: p<0.05 (Veh + cold vs. Cap + cold), $$: p<0.01 (Veh + cold vs. Cap + cold), #: p<0.05 (Cap + cold vs. Cap), ##: p<0.01 (Cap + cold vs. Cap). Veh: vehicle for capsaicin, Cap: capsaicin.

Many cells expressed pERK-LI after noxious heat (50°C) stimuli of the lateral facial skin and also showed NeuN immunoreactivity indicating that they were neuronal in nature (Fig. 4A–C). These pERK-LI cells were observed in Vc and C1-C2 within 2 min after cessation of heat stimulus (50°C) and peaked at 4 min, and subsequently declined in number (Fig. 4D). This time-course change in pERK-LI cell expression in Vc was similar to that described by Noma et al. [30]. There was a large number of pERK-LI cells in Vc and C1-C2 following non-noxious (40°C) as well as noxious (45 and 50°C) heat stimuli of the lateral face in both vehicle-treated and capsaicin-treated rats, as illustrated in Fig. 5. These pERK-LI cells were especially apparent in the superficial laminae of Vc and C1-C2. Typical rostro-caudal distribution patterns of the pERK-LI cells expressed by various heat stimuli are shown in Fig. 5A–F. These pERK-LI cells were greatest in number at -2880 μm caudal to the obex at each stimulus temperature (Fig. 5G–L). The number of pERK-LI cells was also significantly increased following progressive increases in the stimulus temperature (Fig. 5M), and was also significantly larger in capsaicin-treated rats compared to vehicle-treated rats. A small number of pERK-LI cells were observed after capsaicin treatment without heat or cold stimulus (Fig. 5M and 6M). We also studied the effect of the intrathecal (i.t.) administration of the MEK inhibitor, PD98059, on ERK phosphorylation in Vc and C1-C2 neurons. The number of pERK-LI cells expressed by a 50°C stimulus in capsaicin-treated rats was significantly smaller in i.t. PD98059-injected rats compared to i.t. vehicle-injected rats (Fig. 5N).

As illustrated in Fig. 6, a large number of pERK-LI cells was also observed in the superficial laminae of Vc and C1-C2 at 4 minutes after cold stimulus of the lateral facial skin in rats with vehicle or capsaicin treatment (Fig. 6A–F). Peak numbers occurred at -2880 μm to the obex, similar to that following heat or mechanical stimulus (Fig. 6G–L). The number of pERK-LI cells significantly increased following progressive decreases in the stimulus temperature from 25 to 5°C, as illustrated in Fig. 6M. The number of pERK-LI cells was also significantly larger in capsaicin-treated rats compared to that of vehicle-treated rats at each stimulus temperature (Fig. 6M). The increment ratio of the number of pERK-LI cells in Vc and C1-C2 following thermal stimuli of the face was much higher in capsaicin-treated rats compared to that in rats with vehicle treatment (heat, vehicle: 50°C/38°C = 162.9%, capsaicin: 50°C/38°C = 209.9%; cold, vehicle: 5°C/38°C = 162.6%, capsaicin: 5°C/38°C = 244.3%).

**Figure 7:**
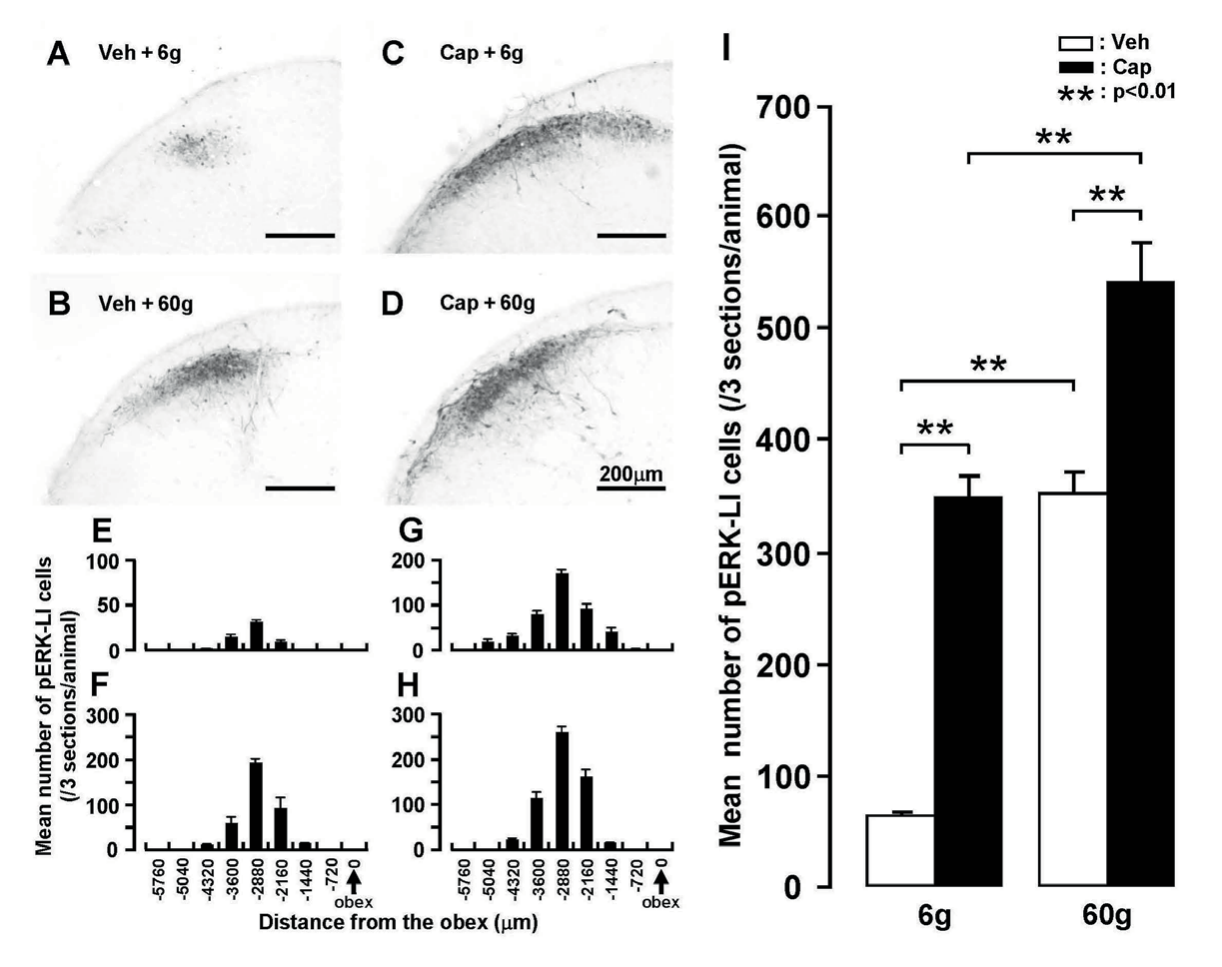
**pERK-LI cells in Vc after non-noxious and noxious mechanical stimuli of the lateral facial skin in rats with capsaicin or vehicle application to the skin**. A-D: Photomicrographas of Vc following no-noxious (6 g) or noxious (60 g) mechanical stimulus of the skin in vehicle- (A and B) or capsaicin-treated (C and D) rats. E-H: Rostro-caudal distribution of pERK-LI cells in Vc and C1-C2 following non-noxious and noxious mechanical stimuli of the skin in vehicle- (E and F) and capsaicin-treated (G and H) rats. M: Mean number of pERK-LI cells in Vc and C1-C2 following non-noxious and noxious mechanical stimuli of the skin in vehicle- (open columns) and capsaicin-treated (solid columns) rats. **: p < 0.01. Veh: vehicle for capsaicin, Cap: capsaicin.

A large number of pERK-LI cells was also observed in the superficial laminae of the Vc and C1-C2 at 4 min after non-noxious (6 g) and noxious (60 g) mechanical stimuli of the facial skin (Fig. 7A–D). Most pERK-LI cells were restricted to the dorso-ventral middle portion of Vc and C1-C2. Dense labeling of fibers intermingled with many pERK-LI cells was also observed in Vc and C1-C2. The number of pERK-LI cells peaked at -2880 μm to the obex in rats receiving mechanical stimuli of the lateral facial skin (Fig. 7E–H). In addition, the number of pERK-LI cells in Vc and C1-C2 following non-noxious (6 g) or noxious (60 g) mechanical stimuli of the lateral facial skin was significantly larger in capsaicin-treated rats compared to that of vehicle-treated rats (Fig. 7I). The increment ratio of the number of pERK-LI cells in Vc and C1-C2 following mechanical stimuli of the face was much higher in vehicle-treated rats compared to that in rats with capsaicin treatment (vehicle: 60 g/6 g = 545.6%, capsaicin: 60 g/6 g = 156.0%), as illustrated in Fig. 7I.

### Effect of peripheral capsazepine administration on ERK phosphorylation in Vc and C1-C2 neurons

**Figure 8:**
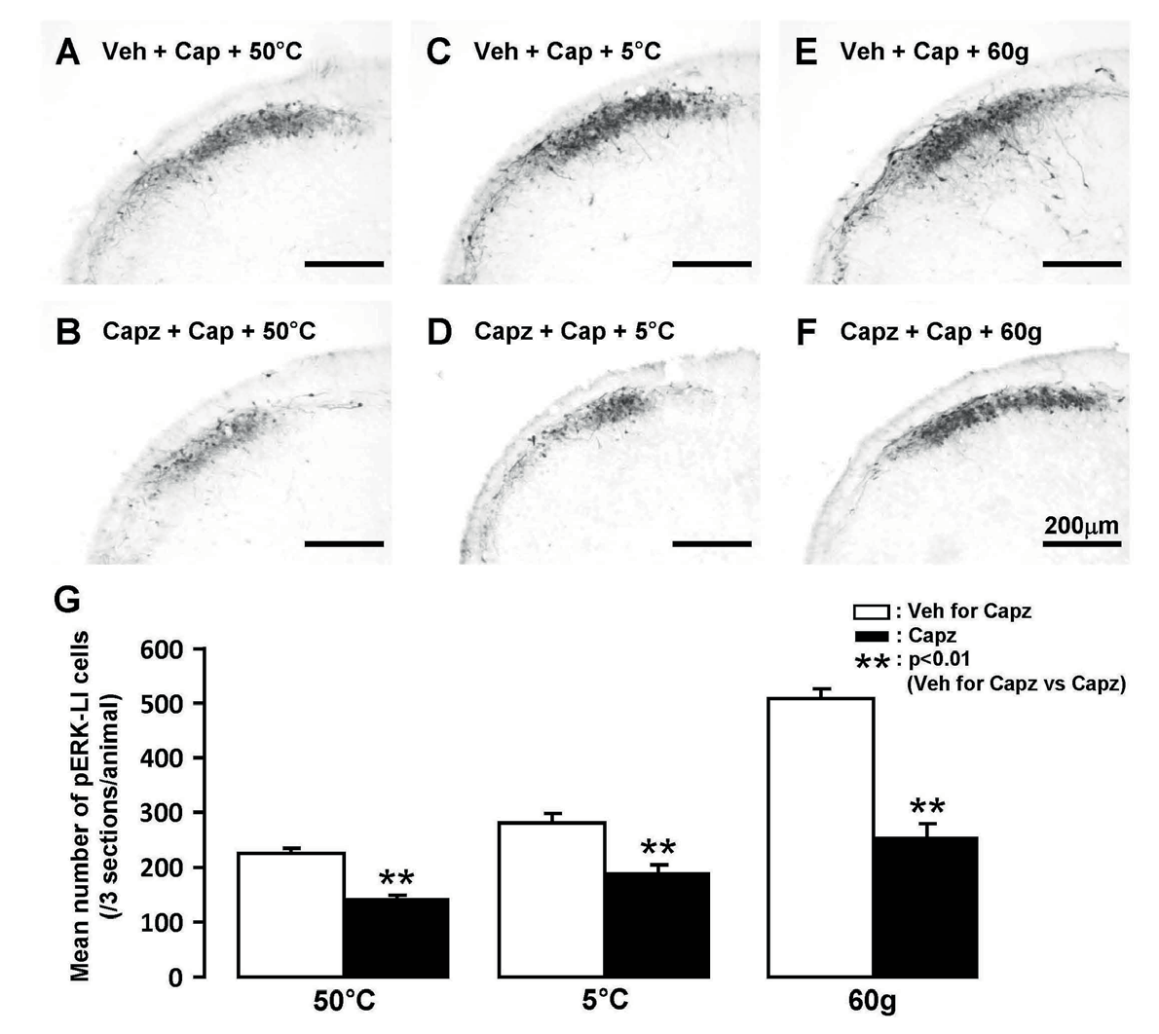
**Effect of subcutaneous capsazepine injection into the facial skin on the noxious heat, cold or mechanical stimulus-induced pERK-LI cells in capsaicin-treated rats**. A and B: Vehicle or capsazepine injection following 50°C stimulation of lateral facial skin in capsaicin-treated rats. C and D: Vehicle or capsazepine injection following 5°C stimulation of the skin in capsaicin-treated rats. E and F: Vehicle or capsazepine injection following 60g stimulation of the skin in capsaicin-treated rats. G: Mean number of pERK-LI cells in Vc and C1-C2 following noxious heat, cold or mechanical stimuli of the skin in capsaicin-treated rats receiving capsazepine (solid columns) or vehicle (open columns) subcutaneously injection. **: p<0.01. Veh for Capz: vehicle for capsazepine, Capz: capsazepine, Cap: capsaicin.

The potent capsaicin antagonist capsazepine (or vehicle) was injected into the lateral facial skin subcutaneously and then the capsaicin patch was placed over the capsazepine-injected skin. Many pERK-LI cells were observed in Vc and C1-C2 after noxious heat, cold and mechanical stimuli of the lateral facial skin in capsaicin-treated rats with subcutaneous vehicle injection (Fig. 8A–F) but the numbers were significantly smaller in capsazepine-injected rats compared to vehicle-injected rats at heat (50°C), cold (5°C) and mechanical (60 g) stimuli, as illustrated in Fig. 8G.

## Discussion

It has been reported that topical application of capsaicin produces a decrease in escape threshold to heating of capsaicin-treated skin [17] as well as neurogenic inflammation resulting in flare formation [31-33]. These findings suggest that the topical administration of capsaicin may directly activate and sensitize the TRPV1 channels in C-fiber and small-diameter Aδ-fiber terminals distributed in the subcutaneous tissues, resulting in an enhancement of TRPV1 channel activity. The present study documented a marked flare formation after capsaicin treatment of the facial skin. The flare was obvious at 5 min after removal of the 1-hour capsaicin patch but not at 60 min after patch-removal, suggesting that extravasations caused by topical capsaicin treatment are transient.

We observed a significant enhancement of the heat, cold and mechanical-induced nocifensive behaviors after topical administration of capsaicin. The ERK phosphorylation following heat, cold and mechanical stimuli of the facial skin was also significantly enhanced in Vc and C1-C2 neurons in the capsaicin-treated rats. The i.t. injection of MEK inhibitor caused significant attenuation of ERK phosphorylation in Vc and C1-C2 neurons and in nocifensive behaviors to heat, cold and mechanical stimuli in the capsaicin-treated rats. Furthermore, peripheral injection of the capsaicin antagonist capsazepine caused significant attenuation of ERK phosphorylation in Vc and C1-C2 neurons to heat, cold and mechanical stimuli in capsaicin-treated skin. These data suggest that both peripheral and central mechanisms are involved in an enhancement of nociceptive behaviors to both thermal and mechanical stimuli of the capsaicin-treated facial skin.

Heat, cold and mechanical stimuli have frequently been used for nocifensive behavioral tests in rat models of peripheral inflammation or nerve injury [34-37]. A decrease in non-noxious mechanical or thermal escape threshold in inflamed rats is thought to reflect mechanical or thermal allodynia [34,36,38]. On the other hand, we evaluated the mechanical or thermal hyperalgesia in rats showing strong nocifensive behavior to a variety of noxious stimuli after peripheral inflammation [34,38]. We observed a significant reduction in escape thresholds to heat or mechanical stimuli of the facial skin and a significant increase in face-scratching frequency to acetone application to the face in capsaicin-treated rats compared to vehicle-treated rats, suggesting that rats with topical capsaicin application to the facial skin had thermal and mechanical allodynia as well as hyperalgesia.

### pERK-LI cells in Vc and C1-C2

ERK is one of the MAP kinase families and is phosphorylated in DH neurons following a variety of noxious stimuli applied to peripheral tissues [29,39]. We have recently reported that the capsaicin injection into various parts of the facial skin causes considerable pERK-LI cell expression in the superficial laminae of Vc and C1-C2 [30]. The pERK-LI cells expressed by capsaicin injection into the whisker pad skin were restricted to the region 2–3 mm caudal to the obex and to the dorso-ventrally middle portion of Vc and C1-C2. A large number of pERK-LI cells was expressed in a restricted portion of Vc and C1-C2 at 2.16–3.6 mm caudal to the obex and in the dorso-ventrally middle portion of Vc and C1-C2 after heat, cold or mechanical stimuli of the lateral facial skin (Figs. 5, 6, 7). The rostro-caudal and dorso-ventral arrangement of pERK-LI cells in the Vc and C1-C2 was slightly expanded after capsaicin treatment, suggesting that the capsaicin treatment affects the somatotopic arrangement of pERK-LI cells expressed by thermal and mechanical stimuli of the face in Vc and C1-C2.

It has also been reported that ERK phosphorylation in dorsal root ganglion (DRG) neurons is intensity-dependent, since their numbers increase following increases in noxious stimulus intensity [25]. Given this finding and the faithful transmission of intensity signaling between primary afferents and second-order spinal and trigeminal neurons [40], it is highly likely that ERK phosphorylation is increased in an intensity-dependent manner in medullary and upper cervical DH neurons. We indeed observed an intensity-dependent increase in the number of pERK-LI cells in Vc and C1-C2 following increases in heat or cold stimulus intensity in vehicle-treated rats as well as in capsaicin-treated rats. A graded increase in the number of pERK-LI cells in Vc following increases in thermal stimulus intensity has also been reported in Fos studies [41-43]. These findings suggest that pERK-LI cells in Vc and C1-C2 are involved in encoding of noxious stimulus intensity.

We also observed that the number of pERK-LI cells following application of heat, cold or mechanical stimuli was greater in capsaicin-treated rats compared to vehicle-treated rats. The increment ratio of the number of pERK-LI cells was also much higher in capsaicin-treated rats compared to vehicle-treated rats in heat and cold stimuli. These findings suggest that the ability to encode the noxious thermal stimuli in Vc and C1-C2 nociceptive neurons is enhanced following topical administration of capsaicin to the facial skin. In the case of mechanical stimuli, the increase in number of pERK-LI cells was much higher in 6 g stimulus compared to 60 g stimulus resulting in the increment ratio of the number of pERK-LI cells in mechanical stimuli was much larger in vehicle-treated rats compared to capsaicin-treated rats. This suggests that capsaicin treatment causes mechano-allodynia in the facial skin as well as thermal and mechanical hyperalgesia. [12,44,45]

It has been reported that ERK is phosphorylated in non-neuronal glial cells in spinal DH 2–21 days after spinal nerve ligation [46]. The ERK phosphorylation may occur in Vc glial cells in our model as described in the previous reports. These suggest that glial cell activation is also involved in an increase in the number of pERK-LI cells in Vc of the rats with capsaicin treatment.

### Effect of MEK inhibitor PD98059 on nocifensive behaviors

It is well documented that the i.t. administration of the MEK inhibitor PD98059 causes a significant reduction of ERK phosphorylation in Vc neurons as well as spinal DH neurons [47]. It has also been reported that the lowering of escape threshold to a variety of noxious stimuli could be attenuated by PD98059 i.t. injection in rats with peripheral nerve injury [46]. We also documented that enhancement of heat-, cold- and mechanical-induced nocifensive behaviors following application of the inflammatory irritant capsaicin to the facial skin was significantly depressed following i.t. injection of PD98059. A number of studies have reported that nociceptive neurons in Vc and C1-C2 are strongly sensitized following peripheral inflammation or nerve injury [21,38,48-50]. The present study has documented that the number of pERK-LI cells is significantly larger in capsaicin-treated rats as compared to vehicle-treated rats. These findings suggest that pERK may be involved in the hyperexcitability of Vc and C1-C2 nociceptive neurons following facial skin capsaicin application, resulting in a strong enhancement of nocifensive behaviors to thermal and mechanical stimuli.

### Effect of peripheral capsaicin receptor blockage on ERK phosphorylation in Vc and C1-C2 neurons

Capsazepine is a potent inhibitor of the capsaicin channel (TRPV1) [51-54]. A number of previous studies have reported that subcutaneous capsazepine injection into the skin causes a significant decrease in the number of nerve fibers expressing the TRPV1 channel protein [51,52]. These findings strongly suggest that subcutaneous injection of capsazepine into the facial skin may cause a marked reduction in the number of small-diameter fibers expressing the TRPV1 channel protein. We observed that the number of pERK-LI cells in Vc and C1-C2 following heat, cold or mechanical stimuli of the capsaicin-treated facial skin was significantly decreased after subcutaneous capsazepine injection into the lateral facial skin. It has been also reported that the subcutaneous capsazepine injection decreases capsaicin-induced flare formation [54]. Since the flare production and the thermal and mechanical noxious stimuli involve small-fiber activation, these observations suggest that TRPV1 channels are functionally interacting with mechanical and cold receptors directly within nerve terminals or indirectly via neurogenic inflammation.

Many Vc and C1-C2 nociceptive neurons are known to receive A- and C-fiber inputs with a variety of modalities from the orofacial regions [55]. It has been also reported that strong orofacial stimulus causes neuroplastic changes in Vc nociceptive neurons, resulting in the central sensitization of Vc nociceptive neurons [56,57]. The blockade of TRPV1 channels may reduce the hyperexcitability of Vc and C1-C2 nociceptive neurons caused by capsaicin. It is likely that the TRPV1 sensitive afferent input can modulate Vc and C1-C2 nociceptive neurons receiving cold and mechanosensitive afferent inputs from the facial skin.

## Conclusion

We observed a marked increase in flare formation, nocifensive behaviors to heat, cold and mechanical stimuli of capsaicin-treated facial skin, and an increase in the number of the pERK-LI cells in Vc and C1-C2 following heat, cold or mechanical stimulus in capsaicin-treated rats. Both peripheral and central mechanisms may be involved in an enhancement of nocifensive behavior to thermal and mechanical stimuli of the capsaicin-treated facial skin. Two possible peripheral mechanisms are proposed based on previous observations and the present results: The TRPV1 may have a direct interaction with cold- and mechano-channels within C- or Aδ-fiber terminals. Topical capsaicin treatment of the facial skin also causes neurogenic inflammation in the capsaicin-treated skin and also is known to sensitize C- or Aδ-primary afferent terminals, resulting in the heat, cold and mechanical hyperalgesia and allodynia in the capsaicin-treated skin via peripheral inflammatory processes.

The other possible mechanism involves central sensitization in Vc and C1-C2 neurons. It is likely that neuroplastic changes may be induced in Vc and C1-C2 neurons by peripheral capsaicin treatment, resulting in the sensitization of those neurons receiving multimodal nociceptive afferent inputs from the facial skin and heat, cold and mechanical allodynia and hyperalgesia.

## Methods

This study was approved by the Animal Experimentation Committee at the Nihon University School of Dentistry. All surgery and animal care were conducted in accordance with the National Institutes of Health Guide for the Care and Use of Laboratory Animals and the guidelines for Institutional Animal Care, and the guidelines of the International Association for the Study of Pain [58]. A total of 226 male Sprague Dawley rats weighing 180–220 g were used for the present study.

### Topical capsaicin administration to the facial skin

Rats were anesthetized with sodium pentobarbital (50 mg/kg, i.p.) and placed on a warm mat (37°C). The lateral face hair was shaved. Capsaicin (3.1 mg: Wako Co. LTD, Japan) was dissolved in a solution of 100% ethanol (62.5 μl) and Tween-80 (65.6 μl) in saline (871.8 μl) to produce a 10 mM capsaicin solution. A solution containing 100% ethanol, 7% Tween-80 and saline (0.07 : 0.08 : 1.00) was used as the vehicle. Then, 100 μl (10 mM) solution of capsaicin or vehicle was soaked in an 8 × 8 mm cotton patch and placed on the surface of the lateral facial skin for 1 hour in each animal.

### Measurement of flare in the facial skin

The capsaicin (n = 10) patch was placed on the surface of the lateral facial skin for 1 hour in rats anesthetized with sodium pentobarbital (50 mg/kg, i.p.). Rats were maintained under anesthesia until perfusion. To measure the flare formation, a 50 mg/kg Evans blue solution (10 mg/ml in saline) was intravenously injected through the femoral vein 4 min before perfusion. Then rats were perfused through the aorta with saline. A facial skin photograph was taken and the area stained with Evans blue was measured using NIH image software at 5 min and 60 min after removal of capsaicin patch.

### Behavioral testing

One hour after capsaicin or vehicle patch placement on the lateral facial skin, the patch was removed. Rats were lightly anesthetized with sodium pentobarbital (40 mg/kg, i.p.) and kept lightly anesthetized. Depth of anesthesia assured, as previously described [27]. Bipolar enamel-coated silver wire electrodes were place in the splenius capitis muscle for EMG recording (inter-electrode distance: 5 – 6 mm).

A heat stimulus (50°C) was applied to the lateral facial skin at the site of the patch placement through a contact heat probe (5 mm in diameter: adaptation temperature for probe = 38°C) and the head-withdrawal latency was measured from the onsets of the heat stimulus and neck EMG activity (cut off latency = 30 s) in lightly anesthetized rats (capsaicin: n = 5, vehicle: n = 5). Mechanical escape threshold at the lateral facial skin site was also measured using von Frey filaments in different groups of lightly anesthetized rats (capsaicin: n = 5, vehicle: n = 5). To evaluate the rat's escape threshold, the von Frey mechanical stimuli were applied to the lateral facial skin at the site of capsaicin or vehicle patch placement in ascending and descending series of trials. Each von Frey stimulation was applied 5 times in each series of trials. The escape threshold intensity was determined when rats moved their heads away from at least 1 of the 5 stimuli. The median threshold intensity was calculated from the values after two ascending and one descending series of trials. For measurement of cold nocifensive behavior, capsaicin and vehicle-treated rats (n = 5 in each group) were lightly anesthetized and acetone socked in the cotton pellet was placed on the surface of the lateral face and the number of face-scratching episodes was counted for 1 min.

### pERK and NeuN immunohistochemistries

In order to define the peak time point of pERK-like immunoreactive (pERK-LI) cell expression induced in the Vc and upper cervical cord by noxious heat stimuli (50°C) of the lateral facial skin in rats receiving topical administration of capsaicin to the skin, pERK immunohistochemistry was carried out in capsaicin-treated rats at different survival times following heating of the facial skin 1 hour after capsaicin patch removal (2, 4 and 10 min, n = 5 each group). Since the number of pERK-LI cells peaked at 4 min after 50°C stimuli in capsaicin-treated rats (see Results), other rats (n = 5 each group) were subsequently perfused at 4 min after mechanical (6 and 60 g), heat (40, 45 and 50°C), cold (5, 15 and 25°C) stimuli. The tip of the thermal probe was 5 mm in diameter, and the rate of temperature change was set at 10°C/s as reported in our previous study [59]. Before application of the thermal stimulus to the facial skin, the surface temperature was adapted to 38°C for 180 seconds. After the adaptation, 5 pulses of heat or cold stimulus were applied to the facial skin (60 s duration with 10 s intervals). We also analyzed the pERK expression in Vc and C1-C2 neurons following 38°C probe placement for 340 s as a baseline stimulus (capsaicin: n = 5, vehicle: n = 5). The mechanical stimulus was also applied to the lateral facial skin with von Frey filaments (1 Hz for 5 min). In addition, pERK immunohistochemistory was carried out in capsaicin-treated rats without thermal stimulation (n = 5).

Rats were perfused through the aorta with 1% paraformaldehyde (500 ml) followed by 4% paraformaldehyde in 0.1 M phosphate buffer (PB, pH 7.4, 500 ml). Vehicle-treated rats (n = 5 in each group) were also tested for mechanical, heat and cold stimuli in pERK expression in Vc and upper cervical spinal neurons, and these rats were perfused as above.

The medulla and C1-C2 of each rat was removed and post-fixed in 4% paraformaldehyde for 3 days at 4°C. The tissues were then transferred to 20% sucrose (w/v) in phosphate-buffered saline (PBS) for several days for cryoprotection. Thirty-micron-thick sections were cut with a freezing microtome and every fourth section was collected in PBS. Free-floating tissue sections were rinsed in PBS, 10% normal goat serum in PBS for 1 hour, and then incubated in rabbit anti-Phospho-p44/42 MAP Kinase (Thr202/Tyr204) Antibody (1 : 1000, Cell Signaling Technology, U.S.A) for 72 hours at 4°C. Next, the sections were incubated in biotinylated goat anti-rabbit IgG (1 : 600; Vector Labs, Burlingame, CA, USA) for 2 hours at room temperature. After washing, the sections were incubated in peroxidase-conjugated avidin-biotin complex (1 : 100; ABC, Vector Labs) for 2 hours at room temperature. They were then washed in 0.05 M Tris Buffer (TB), and next incubated in 0.035% 3,3'-diaminobenzidine-tetra HCl (DAB, Sigma Co. LTD, Tokyo), 0.2% nickel ammonium sulfate, and 0.05% peroxide in 0.05 M TB (pH 7.4). The sections were then washed in PBS, serially mounted on gelatin-coated slides, dehydrated in a series of alcohols (from 50 to 100%) and cover slipped.

The pERK-LI cells were drawn under a light microscope (objective:10× or 20×) with an attached camera lucida drawing tube (Neurolucida 2000 MicroBrightField. Colchester. UT, U.S.A). The number of pERK-LI cells was counted from every 6^th ^section. The total number of pERK-LI cells from 3 of every 6^th ^section was calculated and the mean number of pERK-LI cells (/3 sections/rat) was obtained from each animal, in order to avoid variability in the number of immunoreactive cells in each section.

Double immunofluorescence histochemistry was also used to determine if the cells expressing pERK-LI expressed a neuronal label. Heat stimuli (50°C) were applied to the lateral facial skin receiving topical administration of capsaicin and 4 min after heating of the face rats were perfused through the aorta with 1% paraformaldehyde (500 ml) followed by 4% paraformaldehyde in 0.1 M phosphate buffer (PB, pH 7.4, 500 ml). Thirty-micron-thick sections were cut and processed for double-labeling immunohistochemistry for pERK and the neuronal label NeuN. Free-floating tissue sections were rinsed in PBS, 10% normal goat serum in PBS for 1 hour, and then incubated in rabbit anti-Phospho-p44/42 MAPK Antibody (1 : 300) and mouse anti-NeuN Antibody (1 : 1000, Chemicon, Temecula, CA) over night at 4°C and secondary antibodies (FITC- and rhodamine-, 1 : 100; Jackson ImmunoResearch, West Grove, PA) conjugated for 1 hour at room temperature in a dark room. Then the sections were washed in PBS 3 times for 5 min. Sections were mounted on slides and cover slipped in PermaFluor (Sigma, U.S.A).

### MEK inhibitor PD98059 injection

The MEK1/2 inhibitor PD98059 was used as the inhibitor for ERK phosphorylation. PD98059 was initially dissolved in 20% DMSO at a concentration of 1 μg/μl (3.7 mM) as stock solution, and then further diluted to 0.1 μg/μl in 10% DMSO for intrathecal (i.t.) injection [54]. A solution of 10% DMSO (in saline) was used as vehicle.

Rats were anesthetized with sodium pentobarbital (50 mg/kg, i.p.) and a p10 polyethylene tube was inserted into the subdural space thought the C4-C5 spinal cord level and the tip of the tube was located near the C1-C2 level. An osmotic pressure pomp was connected to the tube and placed under the dorsal skin. PD98059 or vehicle was injected at a flow rate of 1.0 μl/hour for 7 days with the osmotic pressure pump. Rats were used for behavioral testing and immunohistochemical analyses, 7 days after i.t. infusion of PD98059 (n = 25) or vehicle (n = 20).

### Capsazepine injection into the lateral face

The capsaicin antagonist capsazepine (10 mM) was dissolved with 100% methanol. Thirty μl of capsazepine (n = 15) or 100% methanol (vehicle for capsazepine; n = 15) was injected into the lateral face subcutaneously 2 min before capsaicin treatment in rats anesthetized with sodium pentobarbital (50 mg/kg, i.p.). Four min after cold (5°C), heat (50°C) or noxious mechanical (60 g) stimuli of the lateral facial skin of the capsaicin-treated rats, rats were perfused through the aorta with 1% paraformaldehyde (500 ml) followed by 4% paraformaldehyde in 0.1 M PB. The rat brain was removed and brain sections were processed for pERK immunohistochemistry.

### Statistical analysis

Results are presented as mean ± SEM. Statistical analysis was performed using analysis of variance (ANOVA) followed by Newman-Keuls test or Student's *t*-test or Welch's *t*-test was also used as appropriate. Differences were considered significant at *p *< 0.05.

## Competing interests

The authors declare that they have no competing interests.

## Authors' contributions

All authors read and approved the final manuscript. KH carried out the experiments and data analysis. JK, MK and YT helped the experiments, data analysis and paper writing. BJS provided data interpretation and helped to finalize the manuscript. YY provided data interpretation. KI conceptualized the hypothesis, designed and supervised the experiments, directed the data analysis, and finalized the manuscript.
